# Supplementary figures and images for: Mendelian randomization analysis reveals a causal effect of Streptococcus salivarius on diabetic retinopathy through regulating host fasting glucose
Source: J Cell Mol Med. 2024 Mar 20;28(7):e18200. doi: 10.1111/jcmm.18200 (PMC10951888; doi:10.1111/jcmm.18200)

Supplementary Fig. 1

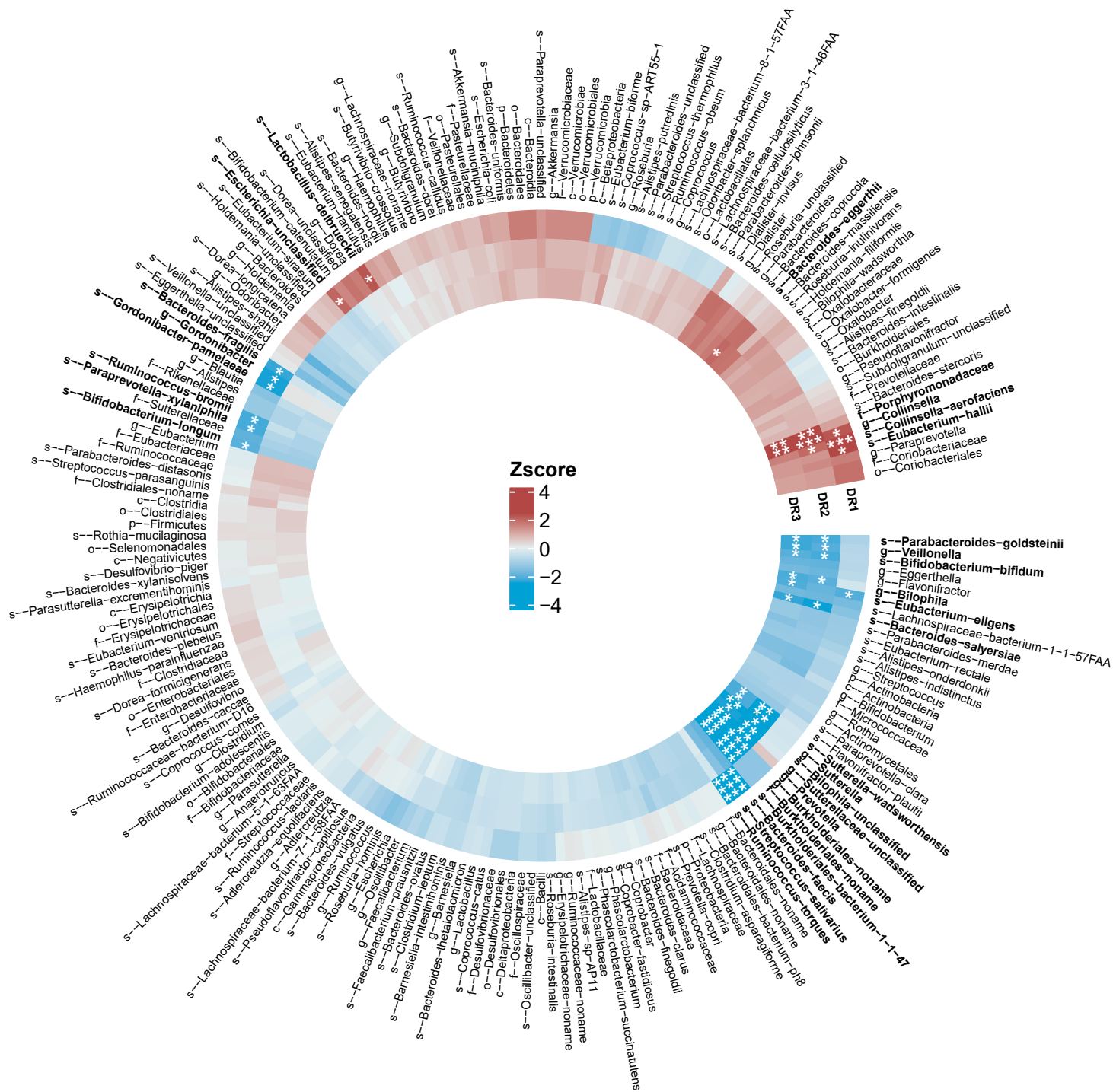

Supplement: Supplementary file 1 — Figure S1. [file JCMM-28-e18200-s003.pdf]

Supplementary Fig. 2

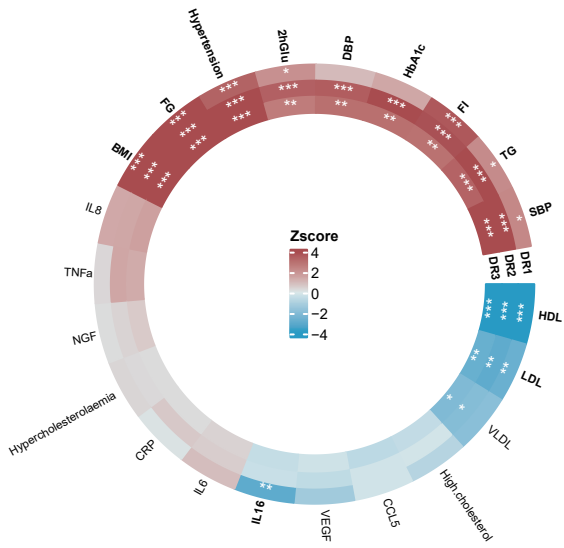

Supplement: Supplementary file 2 — Figure S2. [file JCMM-28-e18200-s001.pdf]
